# Relationship of Thyroid Volume and Function with Carotid and Femoral Intima-Media Thickness in Euthyroid People Aged 18–65 Taking into Account the Impact of Diabetes, Hypertension, and Excess Body Mass

**DOI:** 10.3390/jcm14020604

**Published:** 2025-01-18

**Authors:** Grzegorz K. Jakubiak, Natalia Pawlas, Mateusz Lejawa, Małgorzata Morawiecka-Pietrzak, Jolanta Zalejska-Fiolka, Agata Stanek, Grzegorz Cieślar

**Affiliations:** 1 Department of Pharmacology, Faculty of Medical Sciences in Zabrze, Medical University of Silesia, Jordana 38 St., 41-800 Zabrze, Poland; n-pawlas@wp.pl (N.P.); mateusz.lejawa@gmail.com (M.L.); 2 Department of Pediatrics, Faculty of Medical Sciences in Zabrze, Medical University of Silesia, 3-go Maja 13-15 St., 41-800 Zabrze, Poland; morawieckam@gmail.com; 3 Department of Biochemistry, Faculty of Medical Sciences in Zabrze, Medical University of Silesia, Jordana 19 St., 41-800 Zabrze, Poland; jzalejskafiolka@sum.edu.pl; 4 Department of Internal Medicine, Metabolic Diseases, and Angiology, Faculty of Health Sciences in Katowice, Medical University of Silesia, Ziolowa 45/47 St., 40-635 Katowice, Poland; astanek@tlen.pl; 5 Department of Internal Medicine, Angiology, and Physical Medicine, Faculty of Medical Sciences in Zabrze, Medical University of Silesia, Batorego 15 St., 41-902 Bytom, Poland; cieslar1@tlen.pl

**Keywords:** thyroid volume, thyrotropin, triiodothyronine, thyroxine, carotid intima-media thickness, femoral intima-media thickness, subclinical cardiovascular dysfunction

## Abstract

**Background/Objectives:** The interrelationship of thyroid volume and function with features of cardiovascular dysfunction has already been investigated but some aspects remain unclear, especially in terms of subclinical cardiovascular dysfunction in euthyroid patients. Intima-media thickness (IMT) measurement in ultrasound B-mode imaging in different vascular beds (most frequently within the common carotid artery) is one of the most important tools for the detection of subclinical atherosclerosis in both clinical practice and research. This article aimed to present the results of our research on the association between the thyroid evaluation parameters and the IMT measured in both the carotid and femoral arteries in euthyroid patients aged 18 to 65 years taking into account the influence of diabetes, hypertension, and excess body mass. **Methods:** We performed a retrospective cross-sectional analysis of data from patients with no thyroid disease who during planned hospitalization underwent thyroid ultrasound, determination of biochemical parameters of thyroid function, and Doppler ultrasound of carotid, vertebral, and lower extremities arteries with IMT measurement. Data from 45 people (females: 57.8%) were analyzed. **Results:** No significant correlations were found between biochemical parameters of thyroid function and IMT parameters. Thyroid volume was found to be significantly correlated with all parameters of the IMT within the superficial femoral artery (*R* = 0.407, *p* = 0.007 for the mean IMT calculated from the left side and the right side) and with minimal IMT within the common femoral artery taken from the left side and the right side (*R* = 0.342, *p* = 0.025). Selected IMT parameters were shown to be significantly higher in patients with arterial hypertension, diabetes, obesity, or metabolic syndrome in comparison to patients without the mentioned comorbidities. Using multiple linear regression, it was confirmed that parameters related to thyroid status do not significantly affect the IMT value. A significant effect of diabetes and, to a lesser extent, hypertension on the IMT value was confirmed, especially in the femoral arteries. **Conclusions:** In the population of euthyroid patients, thyroid volume correlates significantly with some parameters of femoral IMT. No significant correlations were found between thyroid biochemical parameters and IMT in both carotid and femoral arteries. A significant influence of diabetes and hypertension on the IMT value was confirmed, especially in the femoral arteries.

## 1. Introduction

Cardiovascular diseases (CVDs) remain one of the most essential health problems worldwide [1]. Atherosclerosis leads to the formation of degenerative structures within arterial walls called atherosclerotic plaques. It may be associated with chronic ischemia of the supplied organ, whereas plaque rupture is associated with sudden blockage of blood flow, which clinically manifests itself as an acute cardiovascular event [2,3,4].

The most important modifiable risk factors for the development of atherosclerosis include, among others, diabetes [5,6], tobacco smoke exposure (active or passive) [7,8], dyslipidemia [9], arterial hypertension [10], low physical activity [11], overweight and obesity [12,13], as well as metabolic syndrome [14]. Mechanisms such as inflammation [15,16], oxidative stress [17,18,19], phenotypic switching of vascular smooth muscle cells [20], or endothelial dysfunction [21,22,23,24], play an important role in the pathogenesis of atherosclerosis.

Identification of patients with subclinical cardiovascular dysfunction seems to be necessary for the implementation of more restrictive control for risk factors, as well as diagnosis of clinically overt CVDs and optimal treatment at an early stage [25]. Measurement of intima-media thickness (IMT) performed by ultrasound imaging (B-mode) is an important tool for the identification of subclinical cardiovascular dysfunction and increased cardiovascular risk, because increased IMT within the arterial wall is an early sign of atherosclerosis development [26,27,28]. The measurement of carotid IMT seems to be most popular in clinical practice and research [29,30], but similarly, IMT can be measured in any vascular bed that can be sufficiently available for ultrasound imaging, such as the femoral arteries [31], radial artery [32], ulnar artery [33], or even the umbilical artery [34].

The literature has paid some attention to the interrelationship between the state of the thyroid gland and the pathogenesis of cardiometabolic diseases [35]. Thyroid function impairment predisposes to increased oxidative stress and inflammation, which is closely related to the pathogenesis of CVDs [36]. Interestingly, the Mediterranean diet, commonly known to be beneficial in terms of CVD incidence, has also been discussed in terms of its beneficial influence on thyroid disease prevention [37].

In our previous articles, we presented the results documenting some relationships of thyroid volume and function with body composition and the diagnosis of metabolic syndrome [38], as well as subclinical cardiovascular dysfunction assessed by the measurement of the ankle-brachial index (ABI), the toe-brachial index (TBI), and toe-pressure (TP) in euthyroid patients aged 18 to 65 [39].

This study aimed to analyze the relationship between the parameters of thyroid assessment and the IMT measured in carotid and femoral arteries in patients aged 18–65 with no abnormalities of thyroid function taking into consideration the impact of diabetes, hypertension, and excess body mass.

## 2. Materials and Methods

### 2.1. Study Participants

The study population included patients hospitalized in the Department of Internal Medicine, Angiology, and Physical Medicine of the Medical University of Silesia in Katowice (Poland) in the period between June 2022 and October 2023 who had undergone thyroid ultrasound and the determination of biochemical parameters of thyroid function and non-invasive cardiovascular assessment. Patients with any acute illness or exacerbation of chronic disease within the month preceding their admission to the hospital, diagnosed thyroid disease (including patients using pharmacological treatment of abnormal thyroid function, even if currently euthyroid), water-mineral balance disturbances, anemia, impaired kidney function, infection, heart failure, atrial fibrillation, diagnosed neoplasm, autoimmune disease, or chronic inflammatory disease were excluded.

The study group was described in detail in our previous publications [38,39].

### 2.2. Laboratory Tests

In terms of thyroid parameters, thyrotropin (TSH) serum concentration as well as free triiodothyronine (FT3) and free thyroxine (FT4) serum concentration were determined in each patient by the electrochemiluminescence assay (ECLIA) using the Elecsys^®^ reagent kits (Roche Diagnostics GmbH, Mannheim, Germany). Auto-antibodies applied in the diagnosis of thyroid diseases and thyroglobulin were not determined.

### 2.3. Thyroid Ultrasound

The thyroid ultrasound was obtained using a Samsung device (RS80 EVO) with a linear probe LA4-18B. The procedure was performed in accordance with the recommendations of the European Thyroid Association [40] and the Polish Ultrasound Society [41]. The same physician performed the examination in each case. Thyroid volume (TV) was calculated by taking into account three measurements obtained for each lobe according to the formula presented in Figure S1.

### 2.4. Arterial Ultrasound with Intima-Media Thickness Measurement

The carotid and vertebral ultrasound was obtained using a Samsung device (RS80 EVO) with a linear probe LA4-18B. The procedure was performed in accordance with the recommendations of the American Society of Echocardiography [42].

The lower extremities ultrasound was obtained using a Samsung device (RS80 EVO). For imaging the abdominal aorta and iliac arteries, a convex probe CA1-7A was used. For imaging the femoral, popliteal, and below-the-knee arteries a linear probe LA3-12A was used.

IMT was measured within the common carotid artery one centimeter below the bifurcation (cIMT) both on the left side (cIMT left) and on the right side (cIMT right), within the common femoral artery one centimeter above the bifurcation (cfIMT) both on the left side (cfIMT left) and on the right side (cfIMT right), and within the superficial femoral artery one centimeter below the bifurcation (sfIMT) both on the left side (sfIMT left) and on the right side (sfIMT rigth). Each measurement was performed three times, and the average value was documented in the patient’s medical history.

Analyzing collected data, taking into account both cIMT right and cIMT left, we presented also the maximal cIMT value (cIMT max), the minimal cIMT value (cIMT min), and the mean IMT value (IMT mean). Analogous data were presented for both cfIMT and sfIMT values.

### 2.5. Statistical Analysis

Numbers and percentages of particular variants were used to present qualitative variables, whereas quantitative variables were presented as mean and standard deviation (variables for which distributions do not differ significantly from normal distribution) or median and interquartile range (variables for which distributions differ significantly from normal distribution). Accordance to the normal distribution of particular continuous variables was tested by the Shapiro–Wilk test, analyzing of distribution parameters, and visual analysis of the histogram.

The significance of differences between subgroups (for example, taking into account comorbidities such as hypertension, carbohydrate metabolism disorders, and excess body weight) was checked by Student’s *t*-test for independent groups (for normally distributed variables) or by the U Mann–Whitney test (for non-normally distributed variables). The significance of differences between the right side and left side in the same parameters was tested by Student’s *t*-test for dependent groups (for normally distributed variables) or by the Wilcoxon test (for not normally distributed variables).

Correlation between different parameters of the IMT and the thyroid parameters (volume and biochemical parameters) was assessed by the Spearman’s rank correlation test.

TIBCO Software Inc. (Palo Alto, CA, USA, 2017) Statistica (data analysis software system, version 13) was used to perform the statistical analysis.

The multiple linear regression analysis was performed using the R programming language within the RStudio environment.

The core of the analysis involved building a multivariable linear regression model to predict IMT as the dependent (predicted) variable. The explanatory variables included in the model were FT3, FT4, sex, BMI, hypertension, and diabetes.

To optimize the model and select the most relevant predictors, stepwise regression was performed using the Akaike Information Criterion (AIC) as the model selection criterion. The AIC is a measure of the relative quality of a statistical model, which considers both the goodness of fit and the complexity of the model. Lower AIC values indicate a better model.

Specifically, the backward elimination method was used. Backward elimination begins with a model that includes all potential predictor variables. The stepwise procedure then iteratively removes the least significant variable, based on its impact on the AIC value. At each step, the model with the lowest AIC is selected, and the process continues until removing additional variables no longer improves the model, meaning the AIC does not decrease further [43].

### 2.6. Ethical Aspects

We obtained confirmation from the Bioethical Committee of the Medical University of Silesia in Katowice that a study involving a retrospective analysis of medical records does not require approval of the Bioethics Committee (6 February 2024, decision no. BNW/NWN/0052/KB/19/24).

## 3. Results

### 3.1. Study Population: General Characteristics and Thyroid Parameters

Data from 45 patients were analyzed (females: 57.8%). Detailed information about the study population was presented in the previous publications [38,39] as well as descriptive statistics for TV, TSH, FT3, and FT4 [38].

### 3.2. Intima-Media Thickness: Descriptive Statistics

In the study population, in 19 patients (42.2%), at least one atherosclerotic plaque was found on the vascular ultrasound examinations performed. In the rest of the study population, no atherosclerotic plaque was found in the performed imaging.

In 35 patients (77.8%), neither cIMT right nor cIMT left exceeded 0.9 mm. In eight patients (17.8%), both cIMT right and cIMT left exceeded 0.9 mm. In one patient (2.2%), cIMT right exceeded 0.9 mm, but cIMT left did not. In one patient (2.2%), cIMT left exceeded 0.9 mm, but cIMT right did not. No significant differences were found between cIMT right and cIMT left (*p* = 0.876) as well as cfIMT right and cfIMT left (*p* = 0.288). sfIMT right was found to be significantly higher than sfIMT left (*p* = 0.014).

Table 1 shows a full description of statistics for cIMT, cfIMT, and sfIMT values in the whole study population. All the presented parameters were not normally distributed.

### 3.3. Correlation Between the Thyroid Function Parameters and Intima-Media Thickness

IMT parameters were not correlated with biochemical parameters of thyroid function (TSH, FT3, FT4).

Full description of the results of the Spearman’s rank correlation test for the relationship between biochemical parameters of thyroid function and IMT values is presented in Table 2.

### 3.4. Correlation Between the Thyroid Volume and Intima-Media Thickness

TV was significantly correlated with all parameters of sfIMT. The strongest correlation was found for sfIMT right (*R* = 0.422), followed by sfIMT mean (*R* = 0.407) and sfIMT max (*R* = 0.391). In terms of cfIMT parameters, a significant correlation was only found between TV and cfIMT min (*R* = 0.342). No significant correlation was found between cIMT parameters and TV.

A full description of the Spearman’s rank correlation test for the relationship between TV and IMT values is presented in Table 3.

The full square correlation matrix including parameters related to thyroid volume and function and IMT values is presented in Figure S2.

### 3.5. Differences Between Subgroups According to the Diagnosis of Atherosclerosis

No significant differences in terms of thyroid volume and function have been found between subgroups according to the diagnosis of atherosclerosis understood as the presence of an atherosclerotic plaque in any vascular bed documented in performed ultrasound imaging. As mentioned in the previous publication [39], no hemodynamically significant atherosclerotic plaque has been found in performed vascular ultrasound imaging (within carotid and vertebral arteries, abdominal aorta, iliac, femoral, popliteal arteries, as well as arteries below-the-knee). In the whole study population, only one patient had diagnosed atherosclerotic CVD and performed percutaneous coronary intervention in the past.

All IMT parameters were significantly higher in patients with atherosclerosis than without atherosclerosis. Although it seems to be obvious per definition, theoretically, it might not have occurred in case of localization of the atherosclerotic plaques in other parts of assessed arteries than the places of IMT measurement. Therefore, we decided to also present differences in IMT between subgroups.

Table 4 shows a complete description of the differences between the subgroups according to the diagnosis of atherosclerosis.

### 3.6. Correlation Between Derivatives of Thyroid Parameters and Intima-Media Thickness

In our previous publication, we presented some significant correlations of additional derivatives from thyroid volume and biochemical parameters (FT3/F4, FT4 × TSH, FT4 × TSH × TV, FT3 × FT4 × TSH × TV) with TBI and TP. The current manuscript is based on data from the same population, so we wanted to present analogous results for IMT parameters to allow comparison of the results for parameters investigated in both publications. Theoretical explanations for the use of such parameters are presented in the previous publication prepared by our team [39].

No significant correlations have been found between IMT parameters and the above-mentioned derivatives from the thyroid parameters.

The results of the Spearman’s rank correlation test for the above-mentioned derivatives from thyroid parameters and the measured vascular parameters are presented in Table 5.

### 3.7. Analysis According to Coexisting Diseases

#### 3.7.1. Arterial Hypertension

The majority of the parameters of cIMT (cIMT right, cIMT mean, and cIMT max) were documented to be significantly higher in patients with arterial hypertension than in patients without arterial hypertension. The median cIMT left and cIMT min were higher in patients with arterial hypertension, but statistical significance was not reached (*p* = 0.054 and *p* = 0.051 appropriately). cfIMT right and cfIMT min were documented to be significantly higher in patients with arterial hypertension than in patients without arterial hypertension, although no significant correlations were found in terms of cfIMT left, cfIMT mean, and cfIMT max. All sfIMT parameters were significantly higher in patients with arterial hypertension when compared to patients without arterial hypertension.

A full description of the differences between subgroups according to the diagnosis of hypertension is presented in Table 6.

#### 3.7.2. Diabetes Mellitus

cIMT right, cIMT mean, and cIMT max were found to be significantly higher in patients with impaired carbohydrate metabolism than in patients with no abnormalities in terms of carbohydrate metabolism. For cIMT left and cIMT min, differences did not achieve statistical significance. All cfIMT and sfIMT parameters were shown to be significantly higher in patients with diabetes or prediabetes than in patients with no diagnosed abnormalities of carbohydrate metabolism.

A full description of the differences between subgroups according to the state of carbohydrate metabolism is presented in Table 7.

#### 3.7.3. Overweight and Obesity

In the study population, cIMT right was significantly higher in overweight or obese patients than in underweight or normal-weight patients. For the other cIMT parameters, the difference did not reach statistical significance. The majority of cfIMT (apart from cfIMT max) parameters were significantly higher in overweight or obese patients than in underweight or normal-weight patients. All sfIMT parameters were significantly higher in patients with BMI ≥ 25.0 kg/m^2^ than in patients with BMI < 25.0 kg/m^2^.

A full description of the differences between subgroups according to BMI category (underweight or normal-weight vs. overweight or obese) is presented in Table 8.

#### 3.7.4. Metabolic Syndrome

All IMT parameters were significantly higher in patients with metabolic syndrome in comparison to patients without metabolic syndrome.

A full description of the differences between subgroups according to the diagnosis of metabolic syndrome is presented in Table 9.

### 3.8. Results of Multiple Linear Regression

In the performed multivariate linear regression analysis, the concentration of free thyroid hormones (FT3, FT4) was not found to have a significant effect on the IMT value both in the CCA and in the femoral arteries (CFA, SFA).

However, a significant influence of diabetes on the IMT value within femoral arteries was confirmed. The selected values are presented in Table 10.

The influence of hypertension diagnosis on the IMT value was clearly weaker than in the case of diabetes. A significant influence of hypertension diagnosis on the sfIMT value on the right side was found, as well as on the minimal value. In the case of some parameters, the influence of hypertension was slightly above the statistical significance limit. The selected values are presented in Table 11.

## 4. Discussion

In the performed analysis, we found no correlation between biochemical parameters of thyroid function (TSH, FT3, FT4) and IMT parameters within common carotid, common femoral, and superficial femoral arteries. Significant correlations have been documented between TV and IMT within superficial femoral arteries as well as between TV and IMT within common femoral arteries (but only the minimal value taken from the right side and left side). Taking into consideration the results from this paper together with the results from our previous publication, it could be concluded that the relationship between thyroid parameters and IMT is weaker than between thyroid parameters and ABI, TBI, and TP. This observation could be an interesting background for further research in this area. Furthermore, the performed analysis of differences in IMT parameters between subgroups according to diagnoses of obesity, metabolic syndrome, diabetes, and arterial hypertension provides the insight that femoral IMT is a valuable tool for subclinical cardiovascular assessment, although it is less frequently used in clinical practice than carotid IMT. In our study population, femoral IMT was shown to be even more affected by cardiovascular risk factors such as arterial hypertension, diabetes, overweight and obesity, and metabolic syndrome than carotid IMT.

The subject of the relationship between thyroid function and IMT value has already been investigated by other researchers. Takamura et al. performed a study in a significantly larger study population (175 men and 468 women) and documented that cIMT negatively correlates with FT4 and positively with logTSH after adjustment for age and sex. However, it should be emphasized that the correlations found were weak (*R* = 0.083, *p* < 0.05 for correlation between log TSH and cIMT in all participants; *R* = 0.093, *p* < 0.05 for correlation between log TSH and cIMT in women analyzed separately; no significant correlation was found for men analyzed separately) [44].

Unal et al. presented the results of an interesting study in which 76 children participated (38 children with subclinical hypothyroidism and 38 euthyroid children). On the one hand, cIMT was shown to be significantly higher in children with subclinical hypothyroidism than in euthyroid children (0.5 vs. 0.4 mm; *p* = 0.001). On the other hand, no significant difference was found in terms of cIMT between children with TSH in the range of 4.2–9.9 mIU/L vs. children with TSH ≥ 10 mIU/L [45]. It is difficult to compare the results obtained in this study to our findings because our study included only euthyroid adults. Asoğlu et al. presented the results obtained from a group of 123 adults (80 patients with subclinical hypothyroidism and 43 euthyroid patients). TSH serum concentration was shown to be significantly correlated with cIMT (*r* = 0.236; *p* = 0.009). In our study, no significant correlation was found between TSH and cIMT, but in our study, there were no patients with subclinical hypothyroidism, whereas in the mentioned study, TSH ≥ 10 *µ*IU/mL was documented in 35 patients [46]. Spilack et al. presented the results of the Brazilian Longitudinal Study of Adult Health (ELSA-Brazil) based on the data obtained from 7551 patients (5077 healthy participants, 1578 diabetic patients, 662 with subclinical hypothyroidism, and 234 diagnosed with both diseases). Although the association of diabetes with increased cIMT was confirmed, no additive effect of subclinical hypothyroidism associated with diabetes over cIMT was detected [47].

Delitala et al. obtained conclusions partially similar to ours. The authors analyzed the data from 5815 subjects not taking thyroid medication, with no overt hyperthyroidism or hypothyroidism. They concluded with the lack of an association of subclinical thyroid dysfunctions with increased IMT or the presence of carotid plaques, although some relationships were found in univariate analysis [48]. Jorde et al. analyzed data from 2034 patients (1856 not taking thyroxine). They did not find any significant relationship between cIMT and TSH in subjects not taking thyroxine, similar to our results. On the other hand, cIMT was documented to be increased in subjects taking thyroxine [49]. Interestingly, Nagasaki et al. found cIMT to be significantly higher in patients with hypothyroidism than in euthyroid patients. Moreover, cIMT was documented to decrease after one year of thyroid function normalization by levothyroxine supplementation [50]. According to another study, TSH serum concentration was shown to be significantly correlated with cIMT in patients with hypothyroidism (*R* = 0.539; *p* < 0.0001). Moreover, levothyroxine therapy for one year was shown to be associated with a decrease in cIMT value [51]. Gao et al. performed a meta-analysis in which it was documented that subclinical hypothyroidism is associated with an increased carotid IMT, which may occur due to elevated thyrotropin (TSH) [52].

In the literature, significantly less data are available on femoral IMT in the context of thyroid function. We did not find any study in which the relationship between femoral IMT and thyroid parameters was analyzed in euthyroid patients. According to Gluvic et al., femoral IMT was shown to be significantly increased in patients with hypothyroidism when compared to controls at baseline, and it was shown to be significantly reduced by treatment. Furthermore, peak systolic flow velocity in the femoral artery was similar to the controls at baseline and significantly decreased with treatment [53]. It was found that radioiodine treatment is associated with significant increase in both carotid and femoral IMT in patients with nodular goiter. A similar but less expressed effect was present in patients with Graves’ disease [54].

Our results regarding the effect of diabetes on IMT thickness are consistent with the results obtained by other researchers. Gateva et al. conducted a study on a group of 461 obese patients, proving that the cIMT value in patients with newly diagnosed diabetes is significantly higher than in people with prediabetes or in people without carbohydrate metabolism disorders [55]. Similarly, according to Rashid, diabetes has a 5.4-fold higher risk of having high cIMT [56]. Kisiel et al. showed that both carotid and femoral IMT are significantly higher in diabetic patients with rheumatoid arthritis compared to controls. Similarly, femoral IMT was significantly higher in diabetic patients with rheumatoid arthritis than in non-diabetic patients with rheumatoid arthritis [57]. Time in the range of 3.9–10.0 mmol/L obtained from continuous glucose monitoring (CGM) was shown to be associated with carotid IMT in a group of patients with type 2 diabetes [58].

Similarly, the effect of hypertension on the IMT value has already been confirmed in other studies. Di Bello presented the results of the study performed on a group of 198 asymptomatic patients with essential hypertension and 67 healthy subjects; cIMT was significantly higher in people with hypertension. Furthermore, cIMT was shown to be significantly correlated with other parameters related to subclinical organ dysfunction, such as pulse pressure and left ventricle mass [59]. Interestingly, according to the study conducted by Dutra et al., the relationship between hypertension and IMT may be bidirectional. In a four-year follow-up, it was found that the cIMT value at the beginning of the observation strongly predicted hypertension occurrence after follow-up in a large multiethnic cohort [60].

### Limitations and Strengths of This Study

The performed study has significant limitations. First of all, the design of the presented research is cross-sectional, so only conclusions about some relationships could be drawn without any insight into cause-and-effect relationships. As mentioned in the previous publications based on the same population, the study group is small, and an analogous study could be repeated on a larger group. It should be emphasized that the auto-antibodies determined in the diagnosis of selected thyroid diseases and thyroglobulin serum concentration were not measured in the current study.

On the other hand, the presented study also has some strengths. Although the study population was small, it should be emphasized that its homogeneity was relatively high because of the exclusion criteria we used. Diagnostics were performed in detail, taking into account thyroid assessment (ultrasound and biochemical parameters) and different methods for non-invasive cardiovascular assessment. All information about patients was based on a current assessment performed during the hospitalization. Furthermore, it should be emphasized that the results described in this paper should be interpreted together with the results described in our previous publications [38,39].

## 5. Conclusions

Analyzing the material collected in this study, we did not find a significant correlation between thyroid function and IMT. Comparing the obtained results with the data from the literature, one should not conclude that such a relationship does not exist. Still, perhaps it was not confirmed in our study due to the too-homogeneous population both in terms of thyroid function (only euthyroid patients, without people with subclinical thyroid dysfunction) and in terms of the cardiovascular system condition (77.8% without thickening of cIMT).

Another conclusion from this study is that measuring IMT in both the common carotid artery and the femoral arteries is a valuable early marker for the development of atherosclerosis. In the material we collected, femoral IMT was even slightly more affected by comorbidities which influence cardiovascular risk than carotid IMT. Therefore, it is worth referring to this parameter, although it is much less popular in clinical practice than carotid IMT.

Although there are currently no recommendations or suggestions for clinical practice to interpret cardiovascular assessment with results of the thyroid function test, the relationship between morphology and function of the thyroid gland and cardiovascular dysfunction seems to be an exciting research direction that may help us better understand some aspects of the pathophysiology of cardiovascular diseases.

## Figures and Tables

**Table 1:** Descriptive statistics of intima-media thickness values in the study population.

Parameter	*n*	Median (Q1; Q3)	Range
cIMT right [mm]	45	0.7 (0.6; 0.83)	0.47–1.2
cIMT left [mm]	45	0.7 (0.6; 0.83)	0.4–1.3
cIMT mean [mm]	45	0.69 (0.62; 0.8)	0.45–1.24
cIMT max [mm]	45	0.73 (0.63; 0.87)	0.5–1.3
cIMT min [mm]	45	0.67 (0.6; 0.8)	0.4–1.2
cfIMT right [mm]	44	0.7 (0.59; 1.04)	0.36–2.5
cfIMT left [mm]	44	0.63 (0.52; 1.09)	0.4–2.5
cfIMT mean [mm]	44	0.67 (0.59; 1.11)	0.38–2.4
cfIMT max [mm]	44	0.72 (0.6; 1.28)	0.4–2.5
cfIMT min [mm]	44	0.6 (0.5; 0.93)	0.36–2.3
sfIMT right [mm]	44	0.53 (0.5; 0.64)	0.3–2.3
sfIMT left [mm]	44	0.5 (0.49; 0.6)	0.3–2.0
sfIMT mean [mm]	44	0.54 (0.49; 0.65)	0.3–1.6
sfIMT max [mm]	44	0.57 (0.5; 0.69)	0.3–2.3
sfIMT min [mm]	44	0.5 (0.47; 0.6)	0.3–1.1

Abbreviations: cIMT—carotid intima-media thickness; cfIMT—common femoral intima-media thickness; sfIMT—superficial femoral intima-media thickness.

**Table 2:** Correlation between the biochemical parameters of thyroid function assessment and intima-media thickness (the results of the Spearman’s rank correlation test).

Parameter	*n*	TSH		FT3		FT4	
		*R*	*p*	*R*	*p*	*R*	*p*
cIMT right	45	−0.064	0.677	0.035	0.818	−0.164	0.282
cIMT left	45	−0.061	0.689	0.069	0.655	−0.083	0.587
cIMT mean	45	−0.071	0.644	0.053	0.732	−0.137	0.37
cIMT max	45	−0.063	0.683	0.043	0.777	−0.177	0.245
cIMT min	45	−0.082	0.595	0.062	0.683	−0.068	0.659
cfIMT right	44	−0.11	0.479	0.231	0.131	−0.092	0.551
cfIMT left	44	−0.291	0.055	0.063	0.687	0.127	0.411
cfIMT mean	44	−0.197	0.199	0.14	0.364	−0.01	0.951
cfIMT max	44	−0.178	0.248	0.14	0.366	−0.058	0.707
cfIMT min	44	−0.24	0.116	0.149	0.334	0.089	0.566
sfIMT right	44	−0.218	0.155	0.094	0.546	−0.068	0.661
sfIMT left	44	−0.191	0.215	−0.062	0.691	−0.105	0.498
sfIMT mean	44	−0.191	0.215	0.029	0.85	−0.068	0.663
sfIMT max	44	−0.201	0.191	0.066	0.669	−0.076	0.623
sfIMT min	44	−0.211	0.169	−0.043	0.784	−0.104	0.504

Abbreviations: cIMT—carotid intima-media thickness; cfIMT—common femoral intima-media thickness; sfIMT—superficial femoral intima-media thickness; TSH—thyrotropin; FT3—free triiodothyronine; FT4—free thyroxine.

**Table 3:** Correlation between thyroid volume and intima-media thickness (the results of the Spearman’s rank correlation test).

Parameter	*n*	Thyroid Volume	
		*R*	*p*
cIMT right	44	0.177	0.249
cIMT left	44	0.206	0.181
cIMT mean	44	0.208	0.175
cIMT max	44	0.147	0.341
cIMT min	44	0.269	0.078
cfIMT right	43	0.272	0.078
cfIMT left	43	0.265	0.086
cfIMT mean	43	0.233	0.132
cfIMT max	43	0.172	0.27
cfIMT min	43	0.342	0.025
sfIMT right	43	0.422	0.005
sfIMT left	43	0.316	0.039
sfIMT mean	43	0.407	0.007
sfIMT max	43	0.391	0.01
sfIMT min	43	0.345	0.023

Abbreviations: cIMT—carotid intima-media thickness; cfIMT—common femoral intima-media thickness; sfIMT—superficial femoral intima-media thickness; significant correlations are marked by red font.

**Table 4:** Differences between patients with atherosclerosis and with no detected atherosclerotic plaque in terms of thyroid volume and function and intima-media values.

Parameter	Patients with Atherosclerosis		Patients with no Detected Atherosclerosis		*p*
	*n*	Mean (SD)/Median (Q1; Q3)	*n*	Mean (SD)/Median (Q1; Q3)	
cIMT right	19	0.9 (0.73; 0.97)	26	0.63 (0.56; 0.7)	<0.001 *
cIMT left	19	0.9 (0.8; 1.0)	26	0.6 (0.6; 0.67)	<0.001 *
cIMT mean	19	0.9 (0.75; 1.03)	26	0.62 (0.57; 0.67)	<0.001 *
cIMT max	19	0.9 (0.8; 1.03)	26	0.67 (0.6; 0.7)	<0.001 *
cIMT min	19	0.87 (0.7; 0.97)	26	0.6 (0.53; 0.67)	<0.001 *
cfIMT right	19	1.1 (0.7; 1.5)	25	0.6 (0.53; 0.7)	<0.001 *
cfIMT left	19	1.27 (0.7; 1.8)	25	0.6 (0.5; 0.63)	<0.001 *
cfIMT mean	19	1.28 (0.75; 1.57)	25	0.6 (0.52; 0.65)	<0.001 *
cfIMT max	19	1.43 (0.8; 1.9)	25	0.63 (0.53; 0.7)	<0.001 *
cfIMT min	19	0.97 (0.63; 1.3)	25	0.53 (0.5; 0.6)	<0.001 *
sfIMT right	19	0.67 (0.53; 0.97)	25	0.5 (0.47; 0.57)	<0.001 *
sfIMT left	19	0.6 (0.5; 0.7)	25	0.5 (0.4; 0.53)	<0.001 *
sfIMT mean	19	0.65 (0.52; 1.07)	25	0.5 (0.44; 0.55)	<0.001 *
sfIMT max	19	0.7 (0.53; 1.13)	25	0.5 (0.47; 0.57)	<0.001 *
sfIMT min	19	0.6 (0.5; 0.7)	25	0.5 (0.4; 0.5)	<0.001 *
TSH [µIU/mL]	19	1.33 (0.53)	26	1.55 (0.81)	0.31 **
FT3 [pg/mL]	19	3.18 (0.54)	26	3.01 (0.56)	0.32 **
FT4 [ng/dL]	19	1.25 (0.15)	26	1.26 (0.2)	0.93 **
TV [mL]	19	12.1 (8.6; 21.5)	25	12.6 (10.8; 15.5)	0.906 *

Abbreviations: cIMT—carotid intima-media thickness; cfIMT—common femoral intima-media thickness; sfIMT—superficial femoral intima-media thickness; TSH—thyrotropin; FT3—free triiodothyronine; FT4—free thyroxine; TV—thyroid volume; (*)—*p*-value according to the U Mann–Whitney test; (**)—*p*-value according to Student’s *t*-test.

**Table 5:** Correlation between selected derivatives from thyroid volume and function parameters, and intima-media thickness (the Spearman’s rank correlation test).

Parameter	FT3/FT4		FT4 × TSH		FT4 × TSH × TV		FT3 × FT4 × TSH × TV	
	*R*	*p*	*R*	*p*	*R*	*p*	*R*	*p*
cIMT right	0.18	0.238	−0.062	0.684	−0.011	0.942	−0.016	0.916
cIMT left	0.111	0.466	−0.018	0.908	0.03	0.848	0.044	0.775
cIMT mean	0.164	0.282	−0.048	0.753	0.003	0.982	0.009	0.956
cIMT max	0.179	0.238	−0.054	0.723	−0.035	0.823	−0.014	0.926
cIMT min	0.116	0.448	−0.038	0.809	0.06	0.701	0.049	0.751
cfIMT right	0.292	0.054	−0.085	0.584	0.038	0.81	0.107	0.496
cfIMT left	0.004	0.98	−0.23	0.133	−0.081	0.606	−0.023	0.883
cfIMT mean	0.157	0.309	−0.158	0.304	−0.039	0.802	0.03	0.848
cfIMT max	0.193	0.209	−0.147	0.342	−0.079	0.614	−0.008	0.958
cfIMT min	0.099	0.521	−0.184	0.231	0.016	0.919	0.08	0.609
sfIMT right	0.142	0.359	−0.203	0.187	0.02	0.901	0.028	0.857
sfIMT left	0.048	0.755	−0.17	0.271	−0.027	0.866	−0.037	0.813
sfIMT mean	0.094	0.544	−0.171	0.267	0.024	0.881	0.022	0.891
sfIMT max	0.12	0.436	−0.183	0.235	0.017	0.913	0.02	0.901
sfIMT min	0.069	0.656	−0.193	0.209	−0.031	0.845	−0.036	0.82

Abbreviations: cIMT—carotid intima-media thickness; cfIMT—common femoral intima-media thickness; sfIMT—superficial femoral intima-media thickness; TSH—thyrotropin; FT3—free triiodothyronine; FT4—free thyroxine; TV—thyroid volume.

**Table 6:** Differences in terms of IMT parameters between patients with and without arterial hypertension.

Parameter	Patients with Hypertension		Patients Without Hypertension		*p*
	*n*	Median (Q1; Q3)	*n*	Median (Q1; Q3)	
cIMT right [mm]	19	0.77 (0.67; 0.9)	26	0.65 (0.57; 0.8)	0.025
cIMT left [mm]	19	0.8 (0.6; 0.93)	26	0.62 (0.6; 0.7)	0.054
cIMT mean [mm]	19	0.75 (0.65; 0.95)	26	0.64 (0.6; 0.75)	0.021
cIMT max [mm]	19	0.8 (0.7; 0.97)	26	0.67 (0.6; 0.8)	0.024
cIMT min [mm]	19	0.73 (0.6; 0.9)	26	0.6 (0.57; 0.7)	0.051
cfIMT right [mm]	18	0.79 (0.67; 1.3)	26	0.65 (0.53; 0.93)	0.031
cfIMT left [mm]	18	0.67 (0.6; 1.43)	26	0.62 (0.5; 0.73)	0.147
cfIMT mean [mm]	18	0.73 (0.6; 1.3)	26	0.63 (0.52; 0.92)	0.069
cfIMT max [mm]	18	0.8 (0.67; 1.43)	26	0.7 (0.53; 1.1)	0.112
cfIMT min [mm]	18	0.65 (0.57; 1.17)	26	0.6 (0.5; 0.7)	0.04
sfIMT right [mm]	18	0.6 (0.57; 0.97)	26	0.5 (0.47; 0.57)	< 0.001
sfIMT left [mm]	18	0.59 (0.53; 0.7)	26	0.5 (0.4; 0.53)	0.001
sfIMT mean [mm]	18	0.62 (0.57; 0.82)	26	0.5 (0.44; 0.55)	< 0.001
sfIMT max [mm]	18	0.65 (0.6; 0.97)	26	0.5 (0.47; 0.57)	< 0.001
sfIMT min [mm]	18	0.57 (0.5; 0.67)	26	0.5 (0.4; 0.5)	0.001

Abbreviations: cIMT—carotid intima-media thickness; cfIMT—common femoral intima-media thickness; sfIMT—superficial femoral intima-media thickness; *p*-value according to the U Mann–Whitney test.

**Table 7:** Differences in terms of IMT parameters between patients with diabetes or prediabetes and patients without abnormalities of carbohydrate metabolism.

Parameter	Patients with Impaired Carbohydrate Metabolism (Diabetes or Prediabetes)		Patients Without Diagnosed Abnormalities of Carbohydrate Metabolism		*p*
	*n*	Median (Q1; Q3)	*n*	Median (Q1; Q3)	
cIMT right [mm]	9	0.9 (0.73; 0.97)	36	0.67 (0.6; 0.8)	0.013
cIMT left [mm]	9	0.9 (0.7; 1.0)	36	0.67 (0.6; 0.8)	0.067
cIMT mean [mm]	9	0.9 (0.75; 0.95)	36	0.67 (0.6; 0.75)	0.033
cIMT max [mm]	9	0.9 (0.77; 1.0)	36	0.7 (0.6; 0.8)	0.019
cIMT min [mm]	9	0.9 (0.7; 0.93)	36	0.6 (0.6; 0.7)	0.051
cfIMT right [mm]	9	1.1 (0.93; 1.5)	35	0.67 (0.53; 0.8)	0.002
cfIMT left [mm]	9	1.46 (1.07; 1.6)	35	0.6 (0.5; 0.7)	0.003
cfIMT mean [mm]	9	1.28 (1.0; 1.5)	35	0.62 (0.55; 0.8)	0.003
cfIMT max [mm]	9	1.46 (1.07; 1.6)	35	0.62 (0.55; 0.8)	0.006
cfIMT min [mm]	9	1.1 (0.93; 1.5)	35	0.6 (0.5; 0.7)	<0.001
sfIMT right [mm]	9	0.8 (0.6; 1.4)	36	0.5 (0.5; 0.6)	0.001
sfIMT left [mm]	9	0.8 (0.53; 1.0)	36	0.5 (0.43; 0.57)	0.002
sfIMT mean [mm]	9	1.07 (0.59; 1.25)	36	0.5 (0.47; 0.59)	<0.001
sfIMT max [mm]	9	1.13 (0.6; 1.7)	36	0.5 (0.5; 0.6)	<0.001
sfIMT min [mm]	9	0.8 (0.53; 0.9)	36	0.5 (0.43; 0.57)	0.004

Abbreviations: cIMT—carotid intima-media thickness; cfIMT—common femoral intima-media thickness; sfIMT—superficial femoral intima-media thickness; *p*-value according to the U Mann–Whitney test.

**Table 8:** Differences in terms of IMT between underweight or normal-weight patients (BMI < 25.0 kg/m^2^) and overweight or obese patients (BMI ≥ 25.0 kg/m^2^).

Parameter	BMI < 25.0 kg/m^2^		BMI ≥ 25.0 kg/m^2^		*p*
	*n*	Median (Q1; Q3)	*n*	Median (Q1; Q3)	
cIMT right [mm]	19	0.63 (0.53; 0.7)	26	0.75 (0.67; 0.87)	0.037
cIMT left [mm]	19	0.63 (0.6; 0.87)	26	0.7 (0.6; 0.83)	0.321
cIMT mean [mm]	19	0.65 (0.57; 0.74)	26	0.75 (0.62; 0.87)	0.087
cIMT max [mm]	19	0.67 (0.6; 0.87)	26	0.8 (0.67; 0.9)	0.101
cIMT min [mm]	19	0.6 (0.53; 0.67)	26	0.7 (0.6; 0.83)	0.091
cfIMT right [mm]	19	0.6 (0.53; 0.73)	25	0.77 (0.67; 1.1)	0.038
cfIMT left [mm]	19	0.6 (0.5; 0.7)	25	0.7 (0.6; 1.1)	0.043
cfIMT mean [mm]	19	0.6 (0.52; 0.92)	25	0.73 (0.62; 1.2)	0.038
cfIMT max [mm]	19	0.63 (0.53; 1.13)	25	0.8 (0.7; 1.43)	0.071
cfIMT min [mm]	19	0.53 (0.5; 0.63)	25	0.67 (0.6; 0.97)	0.016
sfIMT right [mm]	19	0.5 (0.4; 0.6)	25	0.6 (0.5; 0.7)	0.005
sfIMT left [mm]	19	0.5 (0.37; 0.5)	25	0.57 (0.5; 0.67)	0.002
sfIMT mean [mm]	19	0.5 (0.4; 0.55)	25	0.57 (0.52; 0.69)	0.002
sfIMT max [mm]	19	0.5 (0.4; 0.6)	25	0.6 (0.53; 0.7)	0.002
sfIMT min [mm]	19	0.5 (0.37; 0.5)	25	0.53 (0.5; 0.6)	0.004

Abbreviations: cIMT—carotid intima-media thickness; cfIMT—common femoral intima-media thickness; sfIMT—superficial femoral intima-media thickness; *p*-value according to the U Mann–Whitney test.

**Table 9:** Differences in terms of IMT parameters between patients with metabolic syndrome and patients without metabolic syndrome.

Parameter	Patients with Metabolic Syndrome		Patients Without Metabolic Syndrome		*p*
	*n*	Median (Q1; Q3)	*n*	Median (Q1; Q3)	
cIMT right [mm]	13	0.83 (0.73; 0.97)	32	0.67 (0.59; 0.79)	0.003
cIMT left [mm]	13	0.8 (0.7; 0.97)	32	0.62 (0.6; 0.75)	0.016
cIMT mean [mm]	13	0.77 (0.74; 0.95)	32	0.65 (0.6; 0.75)	0.006
cIMT max [mm]	13	0.83 (0.8; 1.0)	32	0.69 (0.6; 0.8)	0.003
cIMT min [mm]	13	0.73 (0.67; 0.93)	32	0.6 (0.59; 0.7)	0.012
cfIMT right [mm]	13	1.1 (0.93; 1.5)	31	0.63 (0.53; 0.7)	< 0.001
cfIMT left [mm]	13	1.43 (0.7; 1.6)	31	0.6 (0.5; 0.7)	< 0.001
cfIMT mean [mm]	13	1.28 (0.8; 1.5)	31	0.6 (0.55; 0.7)	< 0.001
cfIMT max [mm]	13	1.43 (0.97; 1.6)	31	0.7 (0.57; 0.76)	< 0.001
cfIMT min [mm]	13	1.1 (0.7; 1.5)	31	0.57 (0.5; 0.63)	< 0.001
sfIMT right [mm]	13	0.7 (0.57; 1.13)	31	0.5 (0.5; 0.6)	< 0.001
sfIMT left [mm]	13	0.67 (0.5; 0.9)	31	0.5 (0.4; 0.57)	0.002
sfIMT mean [mm]	13	0.7 (0.57; 1.1)	31	0.5 (0.45; 0.57)	< 0.001
sfIMT max [mm]	13	0.7 (0.6; 1.4)	31	0.5 (0.5; 0.6)	< 0.001
sfIMT min [mm]	13	0.67 (0.5; 0.8)	31	0.5 (0.4; 0.57)	0.002

Abbreviations: cIMT—carotid intima-media thickness; cfIMT—common femoral intima-media thickness; sfIMT—superficial femoral intima-media thickness; *p*-value according to the U Mann–Whitney test.

**Table 10:** Results of multiple regression analysis regarding the influence of diabetes diagnosis on individual IMT values in different vascular beds.

Parameter	β	95% CI	*p*
cIMT max	0.06	−0.03, 0.15	0.2
cfIMT right	0.23	0.00, 0.47	0.052
cfIMT left	0.30	0.06, 0.53	0.017
cfIMT mean	0.30	0.08, 0.52	0.008
cfIMT max	0.30	0.04, 0.56	0.027
cfIMT min	0.25	0.06, 0.45	0.012
sfIMT right	0.33	0.20, 0.45	<0.001
sfIMT left	0.18	0.06, 0.29	0.003
sfIMT mean	0.25	0.15, 0.34	<0.001
sfIMT max	0.38	0.23, 0.53	<0.001
sfIMT min	0.12	0.05, 0.18	<0.001

Abbreviations: CI: confidence interval; β: beta coefficient; *p*: *p*-value. β with 95% CI was calculated to assess the association with the outcome variable, adjusted for FT3, FT4, sex, BMI, hypertension, and diabetes.

**Table 11:** Results of multiple regression analysis regarding the influence of arterial hypertension diagnosis on individual IMT values in different vascular beds.

Parameter	β	95% CI	*p*
cIMT right	0.08	−0.03, 0.18	0.14
cIMT left	0.11	−0.01, 0.23	0.076
cIMT mean	0.10	0.00, 0.21	0.054
cIMT max	0.10	−0.02, 0.22	0.10
cIMT min	0.09	−0.02, 0.19	0.093
cfIMT right	0.25	−0.07, 0.57	0.12
cfIMT min	0.20	−0.07, 0.46	0.14
sfIMT right	0.20	0.04, 0.36	0.018
sfIMT mean	0.12	−0.01, 0.25	0.070
sfIMT max	0.14	−0.06, 0.34	0.2
sfIMT min	0.09	0.01, 0.18	0.034

Abbreviations: CI: confidence interval; β: beta coefficient; *p*: *p*-value. β with 95% CI was calculated to assess the association with the outcome variable, adjusted for FT3, FT4, sex, BMI, hypertension, and diabetes.

## Data Availability

The data presented in this study are available upon request from the corresponding author.

## Supplementary Materials

The following supporting information can be downloaded at https://www.mdpi.com/article/10.3390/jcm14020604/s1, Figure S1: Formula for calculating thyroid volume (TV) based on ultrasound measurements; Figure S2: The full square correlation matrix including parameters related to thyroid volume (TV) and function and intima-media thickness (IMT) values.

## Abbreviations

ABI	ankle-brachial index
BMI	body mass index
cIMT	carotid intima-media thickness
cIMT right	carotid intima-media thickness measured on the right side
cIMT left	carotid intima-media thickness measured on the left side
cIMT max	maximal carotid intima-media thickness value (taken from right side and left side values)
cIMT min	minimal carotid intima-media thickness value (taken from right side and left side values)
cIMT mean	mean carotid intima-media thickness value (taken from right side and left side values)
cfIMT	common femoral intima-media thickness
cfIMT right	common femoral intima-media thickness measured on the right side
cfIMT left	common femoral intima-media thickness measured on the left side
cfIMT max	maximal common femoral intima-media thickness value (taken from right side and left side values)
cfIMT min	minimal common femoral intima-media thickness value (taken from right side and left side values)
cfIMT mean	mean common femoral intima-media thickness value (taken from right side and left side values)
CVD	cardiovascular disease
FT3	free triiodothyronine
FT4	free thyroxine
sfIMT	superficial femoral intima-media thickness
sfIMT right	superficial femoral intima-media thickness measured on the right side
sfIMT left	superficial femoral intima-media thickness measured on the left side
sfIMT max	maximal superficial femoral intima-media thickness value (taken from right side and left side values)
sfIMT min	minimal superficial femoral intima-media thickness value (taken from right side and left side values)
sfIMT mean	mean superficial femoral intima-media thickness value (taken from right side and left side values)
TBI	toe-brachial index
TP	toe pressure
TSH	thyrotropin
TV	thyroid volume
