# Supplementary figures and images for: Relationship of Thyroid Volume and Function with Carotid and Femoral Intima-Media Thickness in Euthyroid People Aged 18–65 Taking into Account the Impact of Diabetes, Hypertension, and Excess Body Mass
Source: J Clin Med. 2025 Jan 18;14(2):604. doi: 10.3390/jcm14020604 (PMC11765623; doi:10.3390/jcm14020604)

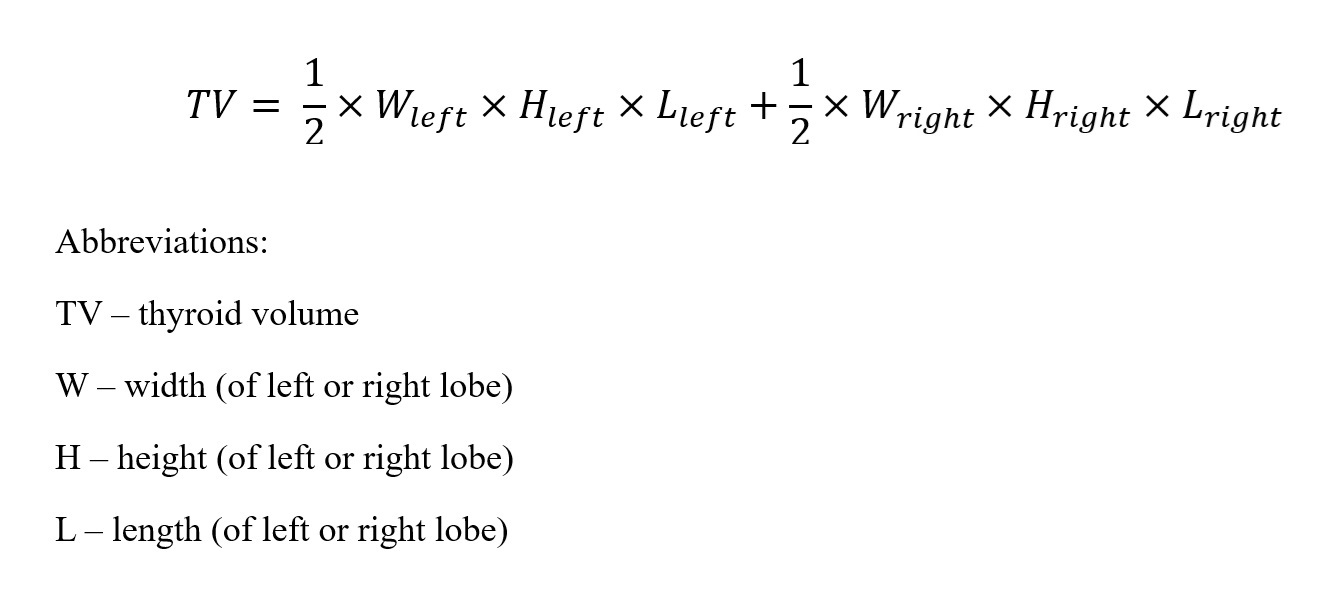

Supplement: Supplementary file 1 [file jcm-14-00604-s001.zip › Figure S1.jpg]

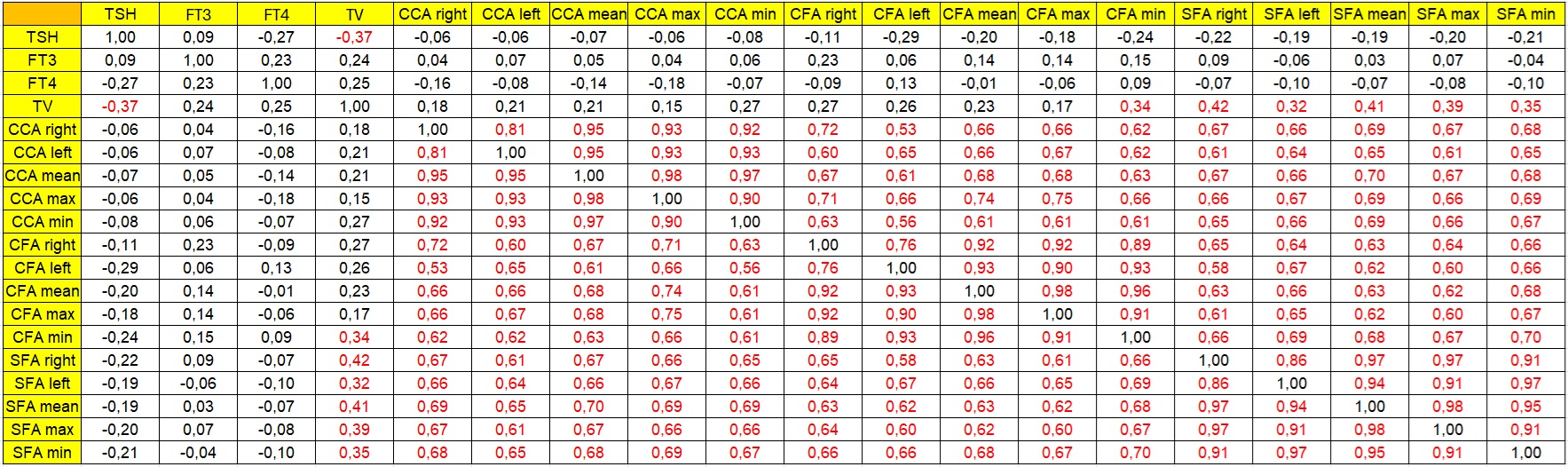

Supplement: Supplementary file 1 [file jcm-14-00604-s001.zip › Figure S2.jpg]
